# A simple bioluminescent method for measuring d-amino acid oxidase activity†
†Electronic supplementary information (ESI) available: Experimental methods, UV-vis data. See DOI: 10.1039/c4cc08145e



**DOI:** 10.1039/c4cc08145e

**Published:** 2014-11-14

**Authors:** T. Spencer Bailey, Micah T. Donor, Sean P. Naughton, Michael D. Pluth

**Affiliations:** a Department of Chemistry and Biochemistry , Institute of Molecular Biology , Materials Science Institute , University of Oregon , Eugene , OR 97403 , USA . Email: pluth@uoregon.edu

## Abstract

d-Amino acid oxidase (DAO) plays important roles in regulating d-amino acid neurotransmitters and was recently identified as a key enzyme integral to hydrogen sulfide production from d-Cys. We report here the development of a simple biocompatible, bioluminescent method for measuring DAO activity based on the highly selective condensation of d-Cys with 6-hydroxy-2-cyanobenzothiazole (CBT-OH) to form d-luciferin.

## Abstract 2

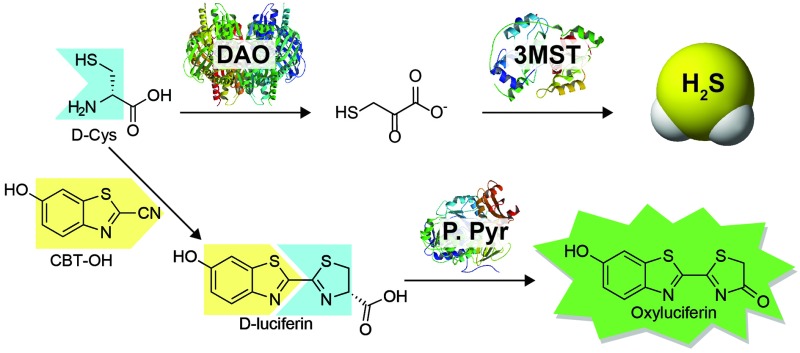
A bioluminescent method for measuring d-amino acid oxidase (DAO) activity was developed based on the luciferin/luciferase reporting platform.

## 

Sulfur-containing compounds constitute a diverse palette of biologically-important molecules that play central roles in signaling and homeostasis. Among these species, hydrogen sulfide (H_2_S) has emerged as an important biological transmitter contributing to signaling processes supporting physiological functions ranging from long term potentiation in the central nervous system to vasorelaxation in the cardiovascular system.^1^ Most prevalent as HS^–^ at physiological pH, sulfide can also be stored in acid-labile, and reductant-labile pools.^2^ Emerging evidence suggests that reductant-labile sulfane sulfur, which includes persulfides (RSSH) and polysulfides (RS(S)_*n*_SR), is an important post-translation modification of Cys residues involved in altered protein function, cellular signalling,^3,4^ and sulfide storage.^5–9^ Most biosynthetic H_2_S is produced enzymatically from Cys and Hcy by the pyridoxal-5-phosphate (PLP) dependent enzymes cystathionine-β-synthase (CBS) and cystathionine-γ-lyase (CSE) as well as l-cysteine aminotransferase (CAT) working together with the PLP-independent enzyme 3-mercaptopyruvate sulfurtransferase (3-MST).^1^ Recent reports now suggests that a fourth enzymatic pathway, primarily active in the cerebellum and kidneys, generates H_2_S from d-Cys.^10^ These findings are supported by the observation that treatment of neuronal tissues with d-Cys results in faster and more efficient H_2_S production than treatment with l-Cys.^10^ H_2_S production from d-Cys relies on d-amino acid oxidase (DAO), which oxidatively deaminates d-amino acids to their corresponding α-keto acids.^11^ Typically involved in neurochemical regulation of d-amino acid neurotransmitters in the brain, DAO metabolizes d-Cys to form 3-mercaptopyruvate (3-MP), which is a substrate for 3-MST (Scheme 1), thus connecting DAO with H_2_S synthesis. Importantly, administration of d-Cys has been shown to elevate sulfane sulfur levels more effectively than l-Cys, suggesting a possible therapeutic potential for d-Cys derived H_2_S.^10^ Despite the importance of DAO in neurochemical regulation and H_2_S production, measuring DAO activity under non-invasive conditions remains a significant challenge.^12^ For example, downstream detection of 3-MP or other α-oxy acids is not a specific assay for DAO activity because these α-oxy acids are also produced by other enzymes. To address this unmet need, we report here a simple bioluminescent method based on firefly luciferase for measuring d-Cys levels and DAO activity.

**Scheme 1:**
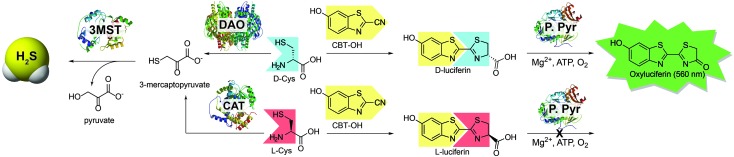
Routes for H_2_S synthesis from 3-MST and the associated strategy for using d-Cys to measure DAO activity using bioluminescence. DAO: d-amino acid oxidase; CAT: l-cysteine aminotransferase; 3-MST: 3-mercaptopyruvate sulfurtransferase; P. Pyr: *Photinus pyralis* (firefly luciferase).

Bioluminescence is a well-studied reporting method readily used for bioimaging with small molecule probes and quantitative measurements in ELISA assays.^13,14^ Because d-luciferin is a common substrate used in bioluminescence studies, we chose to use d-Cys as a substrate in our investigations because it can be metabolized by DAO but can also react with 6-hydroxy-2-cyanobenzothiazole (CBT-OH) to form d-luciferin. Similar condensations of 1,2-aminothiols with 2-cyanobenzothioazoles have been previously applied as a template for polymer aggregation to monitor free Cys and homocysteine,^15^ to monitor caspase activity in the presence of peroxide,^16^ and to develop protein labeling strategies for genetically encoded 1,2-aminothiol residues in proteins^17,18^ allowing for fluorescent and colorimetric imaging in live cell assays. Based on this reactivity and because d-luciferin generates a bioluminescent signal when metabolized by firefly luciferase, we envisioned that treatment of DAO with a known concentration of d-Cys, followed by quenching of unreacted d-Cys with CBT-OH to generate d-luciferin, would provide a simple method for measuring DAO activity.

For this strategy to be biologically compatible, the reaction of CBT-OH with internal Cys residues in proteins or peptides must be reversible to ensure that CBT-OH is not scavenged by GSH or other cellular nucleophiles. Additionally, CBT-OH must react quickly and irreversibly with free d-Cys to generate the d-luciferin product. To establish this feasibility, we investigated the reversible addition of thiols to CBT-OH by ^1^H NMR spectroscopy using *N*-acetyl cysteine (NAC) as a substrate. Because NAC lacks a free amine group, nucleophilic addition to the electrophilic CBT-OH generates an imidothioate that cannot cyclize to form the luciferin product. Treatment of CBT-OH with NAC resulted in complete consumption of the CBT-OH starting material and generation of new aromatic peaks in the ^1^H NMR spectrum, indicating complete reaction of CBT-OH with NAC (Fig. 1b and c). Addition of one equivalent of Cys to the CBT-OH–NAC reaction mixture immediately generates new aromatic peaks as well as a characteristic peak at 5.4 ppm corresponding to luciferin (Fig. 1d). Equilibration of the reaction mixture results in complete conversion of the NAC-adduct to the luciferin product, confirming the reversibility of NAC addition (Fig. 1e). Further treatment of the luciferin product with NAC failed to produce changes in the ^1^H NMR spectrum, thus confirming the irreversibility of Cys addition. When GSH is used in place of NAC the conversion of CBT-OH to luciferin is slower, however, the luciferin product remains the primary reaction product (Fig. S3, ESI†). These results establish that internal thiols in peptides or proteins should not interfere in the detection of Cys by CBT-OH.

**Fig. 1:**
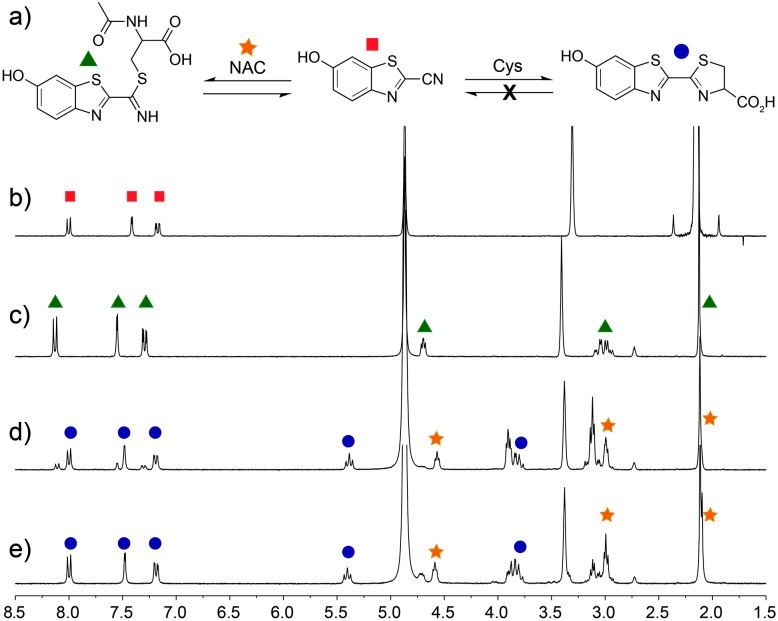
Selectivity of CBT-OH for Cys. (a) CBT-OH reacts reversibly with non-Cys thiols, but irreversibly with Cys. ^1^H NMR spectra (500 MHz, *d*_*6*_-DMSO–D_2_O–CD_3_OD mixture, room temperature) of (b) CBT-OH; (c) after equilibration with 1 equiv. of *N*-acetyl cysteine (NAC); (d) immediately after addition of Cys; and (e) after equilibration.

Having demonstrated that CBT-OH forms luciferin in the presence of multiple thiols, we next investigated whether the condensation rate of CBT-OH with Cys is fast enough to be a viable bioassay method. To measure the rate of reaction of CBT-OH with Cys, we treated CBT-OH with different concentrations of excess Cys under pseudo first-order conditions and monitored the reaction by UV-vis spectroscopy (Fig. S1, ESI†). Based on these kinetic experiments, we determined that the reaction is first-order in Cys and determined an overall second-order rate constant of 14.9 M^–1^ s^–1^ for the reaction (Fig. S2, ESI†), which is similar to the previously reported value of 9.19 M^–1^ s^–1^ for 1,2-aminothiol condensations with cyanobenzothiazoles.^17^ This condensation is almost 200 times faster than the commonly-used azide/cycloalkyne click reaction (7.6 × 10^–2^ M^–1^ s^–1^),^19^ suggesting that the CBT-OH/Cys condensation reaction is kinetically viable for bioconjugation. Having investigated the reaction conditions under pseudo first order conditions, we further explored the suitability of the system for measuring d-Cys concentrations by treating 100 μM CBT-OH with sub-stoichiometric amounts of d-Cys and analyzing the resulting bioluminescent signal. Condensation of CBT-OH with d-cysteine enables us to detect changes in bioluminescent signal at resulting luciferin concentrations as low as 293 nM. After incubation for 1 hour to allow for conversion of CBT-OH to d-luciferin, introduction of luciferase resulted in a bioluminescent response that linearly correlated with d-Cys concentration (Fig. 2b).

**Fig. 2:**
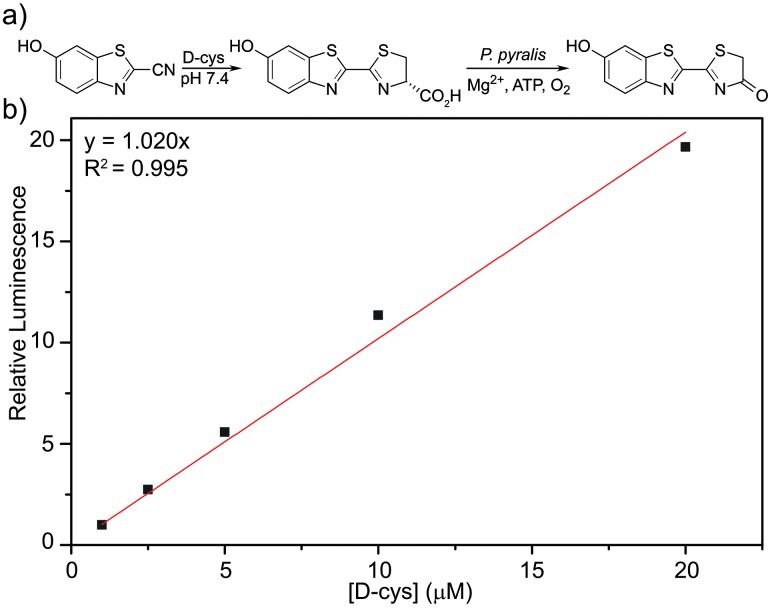
(a) Detection of d-Cys by condensation with CBT-OH followed by treatment with *P. pyralis* generates a bioluminescence response; (b) Bioluminescent response measured with varying concentrations of d-Cys incubated with 100 μM CBT-OH for 1 h followed by addition of 0.1 mg mL^–1^ *P. pyralis*. The bioluminescence was integrated at 560 nm for 45 min in 10 mM Mg^2+^, 2 mM ATP, pH 7.4 tris buffer (50 mM), 37 °C.

Based on the linear bioluminescence response with d-Cys concentration, we next used the CBT-OH system to measure DAO activity. Because DAO metabolizes d-Cys to 3-MP, introduction of an excess of CBT-OH at different time points in the reaction of DAO with d-Cys should convert any remaining, unreacted d-Cys to d-luciferin, which can then be measured by addition of luciferase. To test this design, 20 μM of d-Cys was incubated with 0.1 mg mL^–1^ DAO, and the reaction was quenched at different time points with an excess of CBT-OH. Addition of luciferase and measurement of the resultant bioluminescence from each sample revealed a rapid decrease in the observed bioluminescence signal, correlating with a decrease in d-Cys concentration upon metabolism by DAO (Fig. 3a, black squares). By contrast, use of l- instead of d-Cys did not generate a bioluminescence response, confirming that the l-luciferin condensation product is not a bioluminescent substrate for luciferase. Similarly, if d-Cys is incubated with CBT-OH in the absence of DAO, the bioluminescence stays constant at each time point, confirming a constant concentration of d-Cys in the absence of DAO (Fig. 3a, blue triangles). To further demonstrate that the developed method was reporting on DAO activity, we treated DAO with sodium benzoate, a competitive inhibitor of DAO, (*K*_i_ = 2.0 × 10^–6^ M)^11^ in the presence of d-Cys and measured the bioluminescent response (Fig. 3b). As expected, a significantly higher concentration of d-Cys remained during the assay, confirming that DAO activity was reduced. We also tested the developed assay to probe the affinity of other d-amino acids for DAO. For example, treatment of DAO with equimolar amounts of d-Cys and d-serine did not change the rate of d-Cys metabolism, suggesting that d-Cys is a better substrate for DAO than d-serine.

**Fig. 3:**
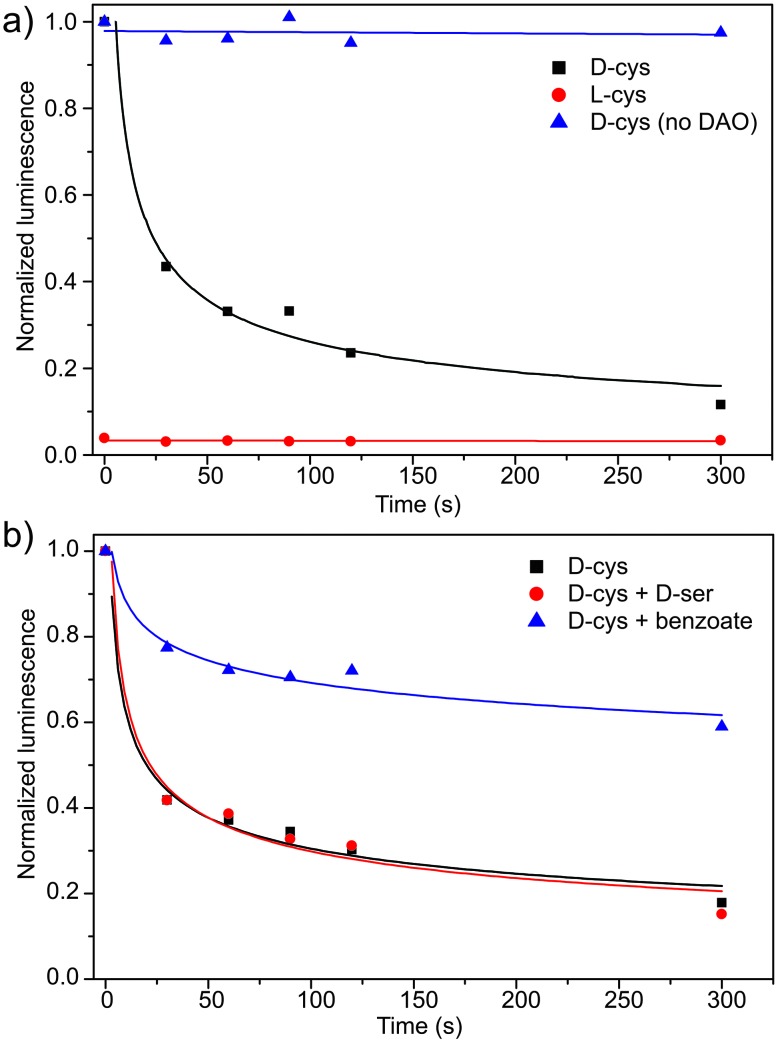
Bioluminescent response of (a) d/l-Cys with CBT-OH in the presence/absence of DAO and (b) d-Cys with the competitive inhibitor benzoate or potential substrate d-serine. Conditions: 20 μM Cys, 0.1 mg mL^–1^ DAO, 40 μM FAD. 50 mM pH 7.4 tris buffer, 37 °C. Competition experiments were performed with 2 μM sodium benzoate or 20 μM d-serine. After incubation, each sample was quenched with 100 μM CBT-OH and imaged with 0.1 mg mL^–1^ *P. pyralis*. at 560 nm for 45 min in 10 mM Mg^2+^, and 2 mM ATP.

In conclusion, the condensation reaction of CBT-OH with Cys provides simple bioluminescent method for measuring d-Cys. We also demonstrated that this condensation reaction can also be used to monitor DAO activity by measuring unreacted D-Cys as a function of time and/or potential DAO inhibitors. Based on the availability of cell lines and animal models that express luciferase enzymes, we envision that this bioluminescent approach for investigating DAO activity and function will provide a useful platform to inform on emerging the roles of DAO in neurochemistry and H_2_S signaling.

We thank Prof. Brad Nolen and his lab for generous use of their plate reader. This work was supported by the NIGMS (R00GM092970) and the University of Oregon (UO). The NMR facilities at the UO are supported by NSF/ARRA (CHE-0923589).
