# Supplementary material for: A simple bioluminescent method for measuring d-amino acid oxidase activity
Source: Chem Commun (Camb). 2014 Nov 14;51(25):5425–8. doi: 10.1039/c4cc08145e (PMC4365669; doi:10.1039/c4cc08145e)
Supplement: Supplementary file 1 [file CC-051-C4CC08145E-s001.pdf]

This journal is © The Royal Society of Chemistry 2014

Supporting Information

A Simple Bioluminescent Method for Measuring D-Amino Acid Oxidase Activity

T. Spencer Bailey, Micah T. Donor, Sean P. Naughton, Michael D. Pluth*

Department of Chemistry and Biochemistry, Institute of Molecular Biology, Materials Science Institute. University of Oregon, Eugene, OR, 97403. USA

Contact: Michael D. Pluth, pluth@uoregon.edu

Table of Contents:

General experimental methods S2
UV-Data S4
NMR Selectivity Data S5

Materials. 2-Cyano-6-hydroxybenzothiazole (CBT-OH), D-serine, D-cysteine•HCl, L-cysteine, N-acetyl cysteine (NAC), sodium beonzoate, adenosine triphosphate (ATP), flavin adenine dinucleotide disodium salt hydrate (FAD), and D-amino acid oxidase (DAO) from porcine kidneys were purchased form commercial sources. Quantilium recombinant lucfierase (*Photinus Pyralis*) was purchased from Promega. Deuterated solvents were purchased from Cambridge Isotope Laboratories and used as received. Tris(hydroxymethyl)aminomethane (tris, Aldrich) was used to make buffered solutions (50 mM tris, pH 7.4) with Millipore water.

Instrumentation. NMR spectra were acquired on a Varian INOVA-500 spectrometer at 25.0 °C. Chemical shifts were measured in parts per million (δ) and were referenced to residual protic solvent resonances. UV-Vis spectroscopic measurements were obtained on a Cary 60, 100, or 300 spectrometer equipped with a Quantum Northwest temperature controllers at 37.0 ± 0.05 °C. Bioluminescence measurements were recorded using a Tecan Safire^2 microplate reader at 37 °C.

NMR Selectivity/Reversibility Experiments. 125 μL of a CBT-OH stock solution (40 mg/mL in d_6-DMSO) was added to 360 μL of an NAC solution (1.1 equiv. NAC, 14 mg/mL in CD_3OD) and allowed to equilibrate ([CBT-OH] = 59 mM, [NAC] = 64 mM). The changes in the NMR spectrum are observed after 15 minutes, and no further changes in the spectrum were observed after 6 hours. After equilibrium was reached, 100 μL of L-cysteine (1 equiv., 34 mg/mL in D_2O) was added, and an NMR spectrum was taken immediately ([CBT-OH] = 49 mM, [NAC] = 53 mM, [L-Cys] = 49 mM). The mixture was allowed to equilibrate over a 24 hour period, and full conversion to L-luciferin observed. An additional 3 equiv. of NAC in CD_3OD were added back into the reaction mixture and no change in the NMR spectrum was observed over a 24 hour period ([CBT-OH] = 22 mM , [NAC] = 93 mM, [L-Cys] = 22 mM).

For the GSH experiments the experimental parameters had to be modified to ensure solubility. 125 μL of the CBT-OH stock solution were added to a mixture of 250 μL of d_6-DMSO and 360 μL of a GSH solution (1.1 equiv. GSH, 26.4 mg/mL in D_2O) and allowed to equilibrate ([CBT-OH] = 39 mM, [GSH] = 43 mM]). The changes in the NMR spectrum were observed after 15 minutes, and no further changes were observed after 1.5 hours as shown in spectrum (d). After equilibration, 100 mL of cysteine solution (1 equiv.) were added ([CBT-OH] = 34 mM, [GSH] = 38 mM, [cysteine] = 34 mM). After 7 hours no further change in the NMR spectrum were observed, presumably due to the complete oxidation of any remaining free thiols. This conclusion is supported by the disappearance of the triplet peak at 4.62 ppm in spectrum (d) and the new appearance of the quartet at 4.75 ppm in spectrum (e), which agrees with the literature value for GSSG. Addition of another 100 mL of cysteine solution and 200 mL of d_6-DMSO results in a simultaneous increase in the luciferin peaks with a corresponding decrease in remaining CBT-OH speaks as seen in spectrum (f) ([CBT-OH] = 25 mM, [GSH] = 28 mM, [cysteine] = 50 mM).

UV-Vis Kinetic Measurements. A 10 mM stock solution of CBT-OH was prepared in DMSO, and a 100 mM solution of L-cysteine was prepared in tris buffer. The CBT-OH solution was diluted to 25 μM in 3 mL of tris buffer. The desired amount of cysteine (250, 375, or 500 μM) was then added to the cuvette, and absorbance measurements were made at 366 nm every 0.05 s for up to 20 min. Each cysteine concentration was repeated in triplicate, and the data were fit directly to an exponential growth curve.

Bioluminescence Detection. CBT-OH (100 μM) and D-cysteine (1-20 μM) were incubated for 1 hour in tris buffer. Luciferin formation was measured by adding 0.1 mg/mL luciferase in tris buffer with 10 mM $MgSO_4$, 2 mM ATP, 0.1 mM DAO, 40 μM FAD. The bioluminescent signal at 560 nm was integrated for 45 min.

DAO Activity Assay. D-cysteine (20 μM) was added to 0.1 mg/mL DAO in Tris buffer with 40 μM FAD. At different time points (0, 30, 60, 90, 120, and 300 s) CBT-OH (100 μM) was added to the solution and the mixture was incubated at 37 °C for 1 hour. Luciferin formation was detected by adding 0.1 mg/mL luciferase in tris buffer (pH 7.4, 50 mM) with 10 mM $MgSO_4$, 2 mM ATP. Bioluminescent signal at 560 nm was integrated for 45 min. Negative controls followed the same method in the absence of DAO or using 20 μM L-cysteine instead of D-cysteine. Inhibition or competition experiments included the addition of either 2 μM sodium benzoate or 20 μM D-serine, respectively.

UV-Vis Data

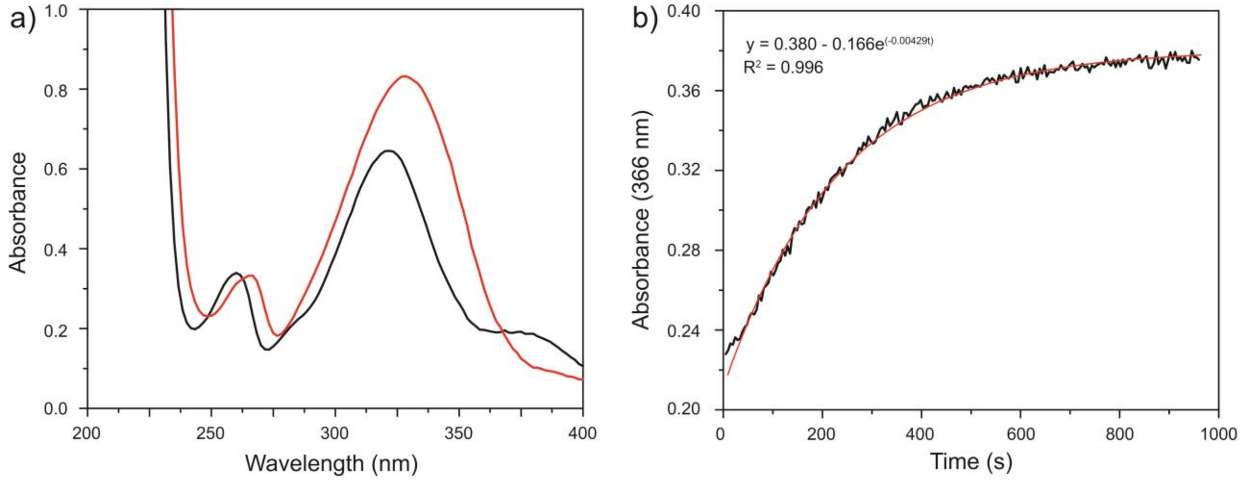

Figure S1. (a) The UV-vis absorbance spectra of 50 μM CBT-OH (black) and D-luciferin (red). (b) The reaction of 25 μM CBT-OH with 375 μM D-cysteine monitored at 366 nm for 15 min with data collected every 0.05 s. The data were fit directly to an exponential growth function to obtain the pseudo-first order rate constant.

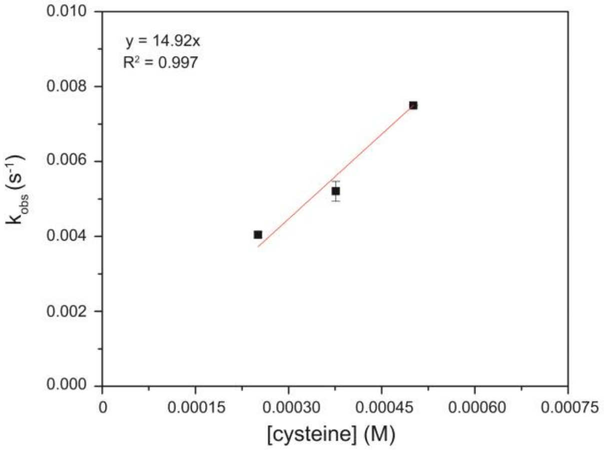

Figure S2. Plot of pseudo-first order rate constant (k_{obs}) with respect to cysteine concentration. Each point is the average of three independent runs. Error bars represent the standard deviation of the data. The slope of the line is the overall second order rate constant for the reaction.

NMR Selectivity Data

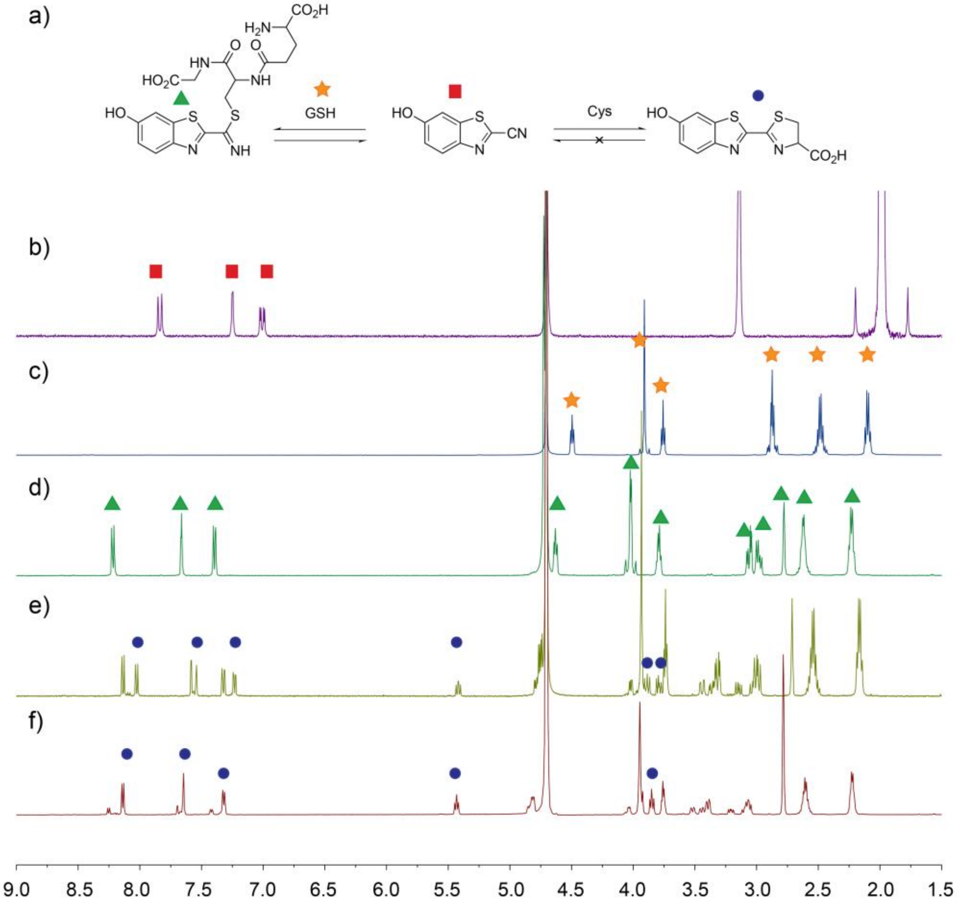

Figure S3. Selectivity of CBT-OH for Cys over GSH. (a) CBT-OH reacts reversibly with GSH and irreversibly with cysteine. ^{1}H NMR spectra (500 MHz, d_6-DMSO/D_2O mixture, room temperature) of (b) CBT-OH and (c) GSH; (d) after 1.5 hr; (e) after 7 hours with 1 equiv of cysteine; (f) after the addition of an additional equivalent of cysteine.
